# Fine root dynamic characteristics and effect on plantation’s carbon sequestration of three *Salix* shrub plantations in Tibetan Plateau alpine sandy land

**DOI:** 10.1002/ece3.7221

**Published:** 2021-02-02

**Authors:** Lingxianzi He, Zhiqing Jia, Qingxue Li, Youyan Zhang, Rina Wu, Jie Dai, Ya Gao

**Affiliations:** ^1^ Institute of Desertification Studies Chinese Academy of Forestry Beijing China; ^2^ Qinghai Gonghe Desert Ecosystem Research Station Qinghai China; ^3^ Research Institute of Forestry Chinese Academy of Forestry Beijing China

**Keywords:** alpine sandy land, carbon sequestration, decomposition, dynamic characteristics, fine root, *Salix*, turnover, vertical distribution

## Abstract

Desertification land in Gonghe Basin of Tibetan Plateau, China accounts for 91.9% of the total land area. Vegetation restoration and reconstruction with desert shrubs in degraded ecosystem are effective ways to prevent and control desertification. However, the evaluation studies of fine root dynamic characteristics of desert shrubs and their contribution to carbon sequestration of plantation are limited. To gain a better understanding of vegetation restoration, the vertical distribution of fine root biomass, fine root decomposition, fine root turnover was investigated, as well as their coupling effect on carbon sequestration of plantation in three desert vegetation. The results estimated that the total decomposition time of fine roots of *Salix cheilophila* (*S. cheilophila*), *Salix psammophila* (*S. psammophila*), and *Salix microstachya* (*S. microstachya*) are 39.00, 27.99 and 35.95 years. Biomass carbon density for three *Salix* plantations ranged from 1.42 to 2.39 t/hm^2^, which showed that three *Salix* plantations in alpine sandy land are an important carbon pool. In addition, fine root biomass carbon density for the three shrub plantations varied significantly. Fine root biomass carbon density for *S. psammophila* reached the largest among the three plantations, which was 1.48 t/hm^2^, accounting for the ratio of 62% of the plantation total biomass carbon density. The results indicated that the root system of *S. psammophila*, especially the fine roots, was very developed, which was conducive to soil water transportation and carbon sequestration. Therefore, *S. psammophila* might be a better species for carbon sequestration of plantation in alpine sandy areas. The carbon input from the fine roots of the three shrub plantations through decomposition and turnover into the plantations accounts for 11.5% to 15.5% of total carbon sequestration of plantations. Therefore, the fine roots dynamics must be considered for long‐term carbon pool estimations in three *Salix* plantations, otherwise the total carbon sequestration of plantations would be underestimated.

## Graphical abstract

The total decomposition time of fine roots of *Salix cheilophila*, *Salix psammophila*, and *Salix microstachya* were significantly larger than that of other regions in desert ecosystem. The increase of N concentration in fine roots of Salix would promote the increase of net primary productivity. The contribution of fine roots to carbon storage of plantation could reach 16% through input soil carbon by dynamic activities such as turnover and decomposition.

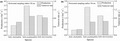

## 1 INTRODUCTION

The area of land degradation in arid and semi‐arid areas in the world reaches 25%, and China is one of the countries most seriously affected by desertification, while the Tibetan Plateau is one of the regions where desertified land is mainly distributed (Zhang et al., 2009). The Gonghe Basin on the Tibetan Plateau is seriously affected by desertification, with 91.9% of the area affected by desertification (Zhang, Gao, et al., 2009). The altitude of Gonghe Basin is between 2,800 and 3,400 m. Compared with other desertified regions, it possessed characteristics, including high altitude, low temperature (1–5.2°C per year), short frost‐free period (64–138 days per year), etc. Therefore, sand areas in Gonghe Basin is one of the most severe natural environmental conditions sand regions with difficulty in vegetation restoration (Li et al., 2019). The establishment of sand‐fixing shrub plantations that has been used extensively in northwestern China since the 1980’s, is one of the most successful and sustainable measures to control desertification and restore degraded ecosystem (Zhang, Li, et al., 2009). Afforestation for desertifcation control has been proposed to effectively promote soil development and enhance carbon (C) sequestration by increasing net primary productivity and root biomass (Su & Zhao, 2003). The magnitude of soil organic carbon (SOC) was associated with the tree species, soil depth, and forest age (Bergner et al., 2004; Deng & Shangguan, 2017; Lai et al., 2016; Li et al., 2019).

The root system that has nutrient absorption and C exchange function is considered as one of the major C pathway to the soil (Ferguson & Nowak, 2011), thus playing an important role in the belowground C sequestration and the biogeochemical cycle (Liao et al., 2014). For vegetation in arid and semi‐arid areas, fine roots (≤2 mm) are main pathways for water and nutrients (Ferguson & Nowak, 2011; Luke McCormack et al., 2013), which possess characteristics of short life cycle, fast turnover and high metabolic activity (Pregitzer et al., 1993; Yuan & Chen, 2010). The dynamic characteristics of fine roots were significant for circulation of materials and energy in terrestrial ecosystems (Clemmensen et al., 2013; Feng et al., 2018; Lai et al., 2017). Although fine roots only account for a small proportion (3%–30%) of the total root biomass in terrestrial ecosystems, it is estimated that a large amount of global net primary production (NPP; 10%–75%) should be consumed to benefit the growth, respiration and turnover of fine roots (Jackson et al., 1997; Yuan & Chen, 2012), and approximately 56% C inputs to the soil through the rapid turnover and decomposition of fine roots (Ruess et al., 2003).

In the forest ecosystem, the amount of carbon and nutrients returned to soil through roots decomposition is markedly larger than that in the above‐ground part (Hobbie et al., 2010). Compared with the temperate ecosystems, fine roots comprise a higher proportion of total plant biomass in the desert ecosystem, which are the dominant pathways through which C enters the soil organic matter (SOM) pool (Jackson et al., 1997; Lai et al., 2016; Plaza‐Bonilla et al., 2014) Therefore, quantification of fine root biomass, production, decomposition, as well as identification of the climatic and other factors related to fine root C, are critical for estimating the role of fine roots as sources of C for modelling carbon sequestration in current and future climates.

Fine root decomposition rate was mainly affected by biological factors such as soil microorganisms and soil animal, as well as abiotic factors such as soil temperature and humidity, which led to concentrating distribution of fine roots on surface soil for seasonal rainfall (Huang et al., 2008; Li et al., 2017). Although previous studies showed that 40%–80% of fine roots were distributed in 0–40 cm (Chang et al., 2012; Lai et al., 2016), time for 95% fine root decomposition need 3.44–11.10 years (Lin et al., 2011), turnover rate of fine roots is 0.02–2.64 year^−1^ (Huang et al., 2008; Jackson et al., 1997; Xu et al., 2013), and dynamic characteristics of fine roots contribute 20%–80% to SOC pool (Lai et al., 2016) in other terrestrial ecosystems, few studies have been done on fine root dynamic characteristics and its effect on plantations carbon sequestration in alpine sandy land. *Salix cheilophila (S. cheilophila), Salix psammophila (S. psammophila), and Salix microstachya (S. microstachya)* were considered as the perfect sand‐fixing species due to their high adaptability to arid and semi‐arid regions with strong winds and lack of precipitation (Yu & Jia, 2014). At present, these three shrub varieties were the dominant sand‐fixing phytocoenoses mainly planted by cuttage in the alpine sand area of Tibetan Plateau. Although their fine root vertical distribution patterns (Lai et al., 2016), water use strategies (Jia et al., 2012), the effects on the soil carbon sequestration and other environmental factors (Yu & Jia, 2014) have been extensively studied, the above studies mainly focused on fine root distribution characteristics for a single species of *Salix*, water use strategies, and the effects of different ages of *Salix cheilophila* on the soil organic carbon and nitrogen storage. The differences in the root systems dynamic characteristics of the three species of *Salix* and their impact on carbon sequestration in plantations is limited. We have used traditional root research methods (soil pillar mining method, litterbag method, soil core method, and forest harvest method) to study the dynamic characteristics (growth, turnover, and decomposition) of fine roots for the three *Salix* species and their contribution to carbon sequestration in plantations.

In this study, we aimed to get the basal data on fine roots’ biomass distribution, turnover, production, and decomposition of three *Salix* shrubs, then tried to explain the variation in carbon sequestration of plantations in Tibetan Plateau alpine sandy land, aiming at offering a theoretical basis for Tibetan Plateau and even global alpine sandy land carbon sequestration. We hypothesized that fine root dynamic characteristics have important contributions to total carbon sequestration of plantations, some of these effects are species‐dependent and environmental factors, such as fine root morphological characteristics, distribution characteristics, frequency of growth and death, the quality of organic carbon exchanged with the soil during the growth and death of fine roots, etc. Thus, the contribution of this is to be tested and calculated in three revegetation *Salix* shrub species. In this paper, our specific objectives were to (a) estimate fine roots’ biomass distribution, turnover, production, and decomposition of *S. cheilophila, S. psammophila, and S. microstachya*; and (b) detect dynamic characteristics and the effect of fine roots on total carbon sequestration of plantations; and (c) expound contribution rate of fine roots in total carbon sequestration of plantations in three *Salix* shrub species.

## 2 MATERIALS AND METHODS

### 2.1 Study area and object

**FIGURE 1:**
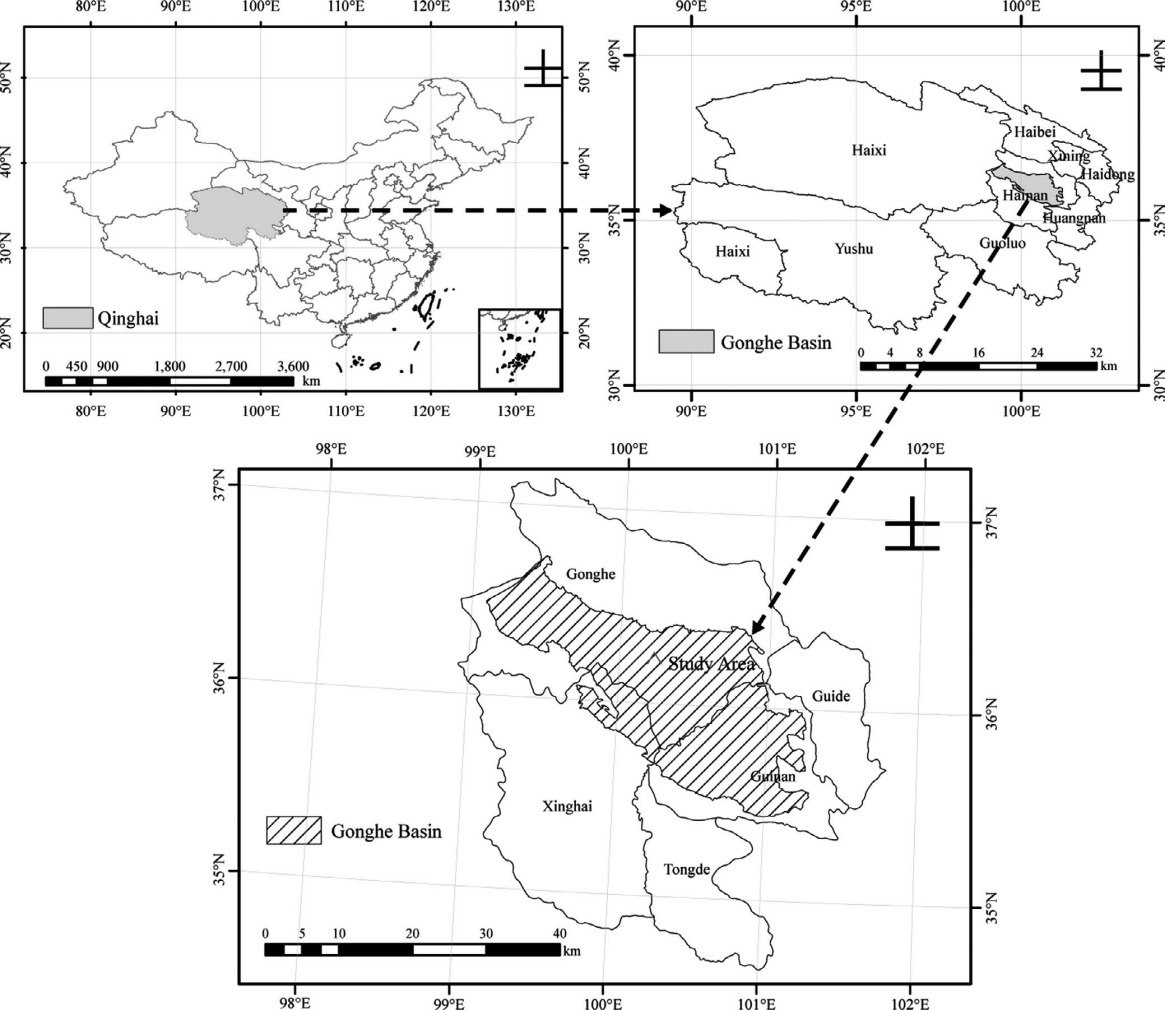
Location of the study area, Gonghe County, Qinghai Province, China (He et al., 2019)

The study was conducted in the desertification combating experimental site of the Qinghai Gonghe Desert Ecosystem Research Station located in northeastern part of the Qinghai–Tibet Plateau (36°03~36°40′N, 99°45′~100°30′E and altitude 2,871 ~ 3,870 m), western China, as Figure 1 illustrates.

The station was constructed by Chinese Academy of Forestry the Desertification Station of Qinghai Province. The climate in the study area is a plateau continental climate with a mean annual temperature, precipitation, and potential evaporation of 1.0~5.2°C, 311.1~402.1 mm and 1716.7 mm, respectively (Li et al., 2018). The mean annual amount of windy days is 50.6 days (up to 97 days). The wind directions are mainly west and northwest, with a mean annual wind speed of 2.7 m/s (up to 40 m/s; Jia et al., 2012). Sand dune and dune slack are alternately in the vegetation restoration area.

In April 1990, the study object of *S. cheilophila*, *S. psammophila*, and *S. microstachya* plantations were planted using cuttage (row spacing of 2 m) in the inter sand dune with sample plot areas 100 m × 100 m, respectively. Fences were used to enclose the vegetation restoration areas for none grazing and fertilization.

### 2.2 Fine root biomass and vertical distribution

Three 20 m × 20 m quadrats of *S. cheilophila, S. psammophila, and S. microstachya* were established in the vegetation restoration zone, respectively. The information of average plant height, basal diameter and crown was recorded, and three standard plants were determined. Fine root biomass was obtained by traditional section soil pillar mining method (Ostonen et al., 2005). Above‐ground parts of standard plants were cut down, and soil columns with a size of 200 cm (horizontal East–West) × 100 cm (horizontal South–North) × 80 cm (vertical depth), were excavated. From top to bottom, every 20 cm was determined as one layer (0~20, 20~40, 40~60, and 60~80 cm), and root samples of different soil depth were obtained every 20 cm from the west to the east. Root samples were kept in valve bags, while impurities, death roots, and those roots with diameter greater than 2 mm, were removed by soil sieve of 0.5 mm mesh. WinRHIZO Tron 2015a was adopted to scan fine roots, and root average diameter, length, surface area, and volume data were obtained. In addition, dry weight after 85°C drying was recorded.

### 2.3 Fine root decomposition rate determination

To determine the decomposition rate, three 5 m × 5 m quadrats were established for *S. cheilophila, S. psammophila, and S. microstachya* in May 2017. Fine roots of three *Salix* shrubs were obtained randomly from surface soil layer (0~20 cm) by digging within quadrats, and then dark, inelastic and necromass root were eliminated in lab. Fine roots were washed and air dried at 85°C to constant weight, some of which was kept for initial chemical composition analysis. Other fine roots were cut into 2 cm in length and mixed respectively that every litterbag (10 cm × 15 cm, with 0.5 mm nylon mesh) was filled with a certain amount (2.000 g). Five plants in the field that have good growth state and similar growth vigor were selected as standard plants, below which the nylon meshes were buried in 30 cm deep holes correspondingly. In May 2017, 75 bags of fine roots for each species were prepared and covered by forest soil and litter in the study area. The buried litterbags were taken out in July and September 2017 and June, August and October in 2018 (15 bags each time), respectively, due to low temperature from November to the following April that relevant experiment was hardly carried out. New roots were removed, and rest of roots were washed and air dried at 85°C to constant weight. The fine roots were obtained through milling and 100‐ mesh sieving for nutrient (C, N, P, K) contents measurement. Total C and N of fine roots were determined using Elemental CHNS analyzer (Vario EL III, Elemental Analyzer System, GmbH, Germany), while total P and K were analyzed with HNO_3_ digestion method using 6300 ICP‐OES (Thermo Scientific, USA). Nonlinear exponential attenuation model (Olson, 1963) was introduced for regression analysis.

### 2.4 Fine root turnover rate and production determination

Dynamic investigations were conducted on the fine root life cycle and turnover characteristics of the plantations every month during the growing season (from June to September). Fine roots were sampled using a steel bucket‐type root auger (8.5 cm in diameter and 25 cm in height) with T‐handle. Horizontal depths of 20 cm and 30 cm from the center of the tree were measured in depths of 0~20, 20~40, and 40~60 cm in the north, southwest, and southeast of the plant. All undisturbed soil cores were installed into the value bag, respectively. Three soil cores collected at the same depth different sample direction were mixed to ensure a good representation of fine roots in each sample. Therefore, 81 soil cores (3 quadrats × 3 vertical depths × 3 horizontal sampling directions × 3 repetitions) were obtained for each sampling of *S. cheilophila*, *S. psammophila,* and *S. microstachya*, 27 soil samples were established for the determination of fine root biomass finally.

All fine roots were manually collected to measure annual fine root production with growth cores of shrub species. All fine root (living and dead) samples were washed with distilled water and then dried at 85°C until dry weight of fine root was obtained. The compartmental flux model method (Vogt et al., 1998) was introduced to calculate the annual NPP and turnover rate of fine roots.

### 2.5 Plantation biomass and soil and vegetation chemical properties measurement

All the above‐ground parts of the three standard shrubs were weighed for fresh weight and the standard branches were randomly selected to obtain the fresh weight of different organs (trunk, bark, branch, leaf). The root system of the standard plant was excavated by the method of section soil column to obtain root samples with different soil depths (referring to the method mentioned above of fine root biomass and vertical distribution). In the plots, soil of per layer was sifted by soil sieve of 0.5 mm mesh to gain all roots. The roots were divided into thick roots (Roots > 5 mm), middle roots (2 mm < Roots ≤5 mm), and fine roots (Roots ≤ 2 mm) with vernier calipers. All standard plant samples were washed with distilled water and then dried at 85°C until dry weight of roots and above‐ground different organs were obtained.

Approximately, 100 g of sieved soil was collected from each of four soil depth intervals within three shrub plots. To have a good representation of SOC in each sample, 3 soil cores collected at the same depth of three shrub plots were mixed. To identify the effect of shrub planting on SOC content, a bare land plot (30 m × 30 m) were selected, where 12 soil cores were randomly collected and then divided into four depth intervals. A total of 144 soil samples (3 shrub plots × 4 depth intervals × 12 replicates) were collected and sieved (pore size < 2 mm), milled ground, and stored in value bags until analysis.

The corresponding formulas for calculating total carbon sequestration of plantations are presented blow.

(1) $$pc_{d}=\sum B_{i}\times c_{i}$$

(2) $$S_{d}=\sum \rho b_{i}\times P_{i}\times D_{i}\;\times k$$

(3) $$T_{d}=P+L+S$$

For the above, *pc_d_* is biomass carbon density of vegetation components, *B*_i_ is vegetation biomass of different components (t/hm^2^), *c_i_* is carbon concentration of vegetation in each component (%), *S*_d_ is soil organic carbon (SOC) density, *ρb_i_* is soil bulk density of layer *i* (g/cm^3^), *P_i_* is SOC concentration in layer *i* (g/kg), *D_i_* is soil thickness of layer *i* (cm), *k* is the unit conversion coefficient, *k* = 0.1; *T_d_* is the total carbon sequestration of plantation (t·hm^−2^·year^−1^), P is the total carbon sequestration of vegetation (t hm^−2^ year^−1^), *L* is the total carbon sequestration of litter (t hm^−2^ year^−1^), and *S* is the total carbon sequestration of soil (t hm^−2^ year^−1^), respectively.

Carbon concentration of soil was determined using the dichromate oxidation method (Walkley & Black, 1934), and carbon concentration of vegetation in each component was determined using Elemental CHNS analyzer (Vario EL III, Elemental Analyzer System, GmbH, Germany).

### 2.6 Statistical analysis

Statistical analyses were conducted using SPSS 20.0 statistical software package (SPSS Inc., Chicago, IL, USA). The differences of the fine root biomass, fine root necromass, fine root production, SOC content, and its fractions among soil layers were compared by one‐way ANOVA test and LSD test. The *t*‐test was used to compare the effect of three shrubs fine root parameters and SOC content on the annual total carbon sequestration of plantation. Four‐way analysis of variance (ANOVA) accompanied by Pearson correlation coefficient analysis method was applied for examining statistical differences among the mass loss and C, N, P, K release of standardized fine root litter at different time intervals. Mean differences were considered significant at *p* < 0.05.

## 3 RESULTS

### 3.1 Fine root biomass vertical distribution

**FIGURE 2:**
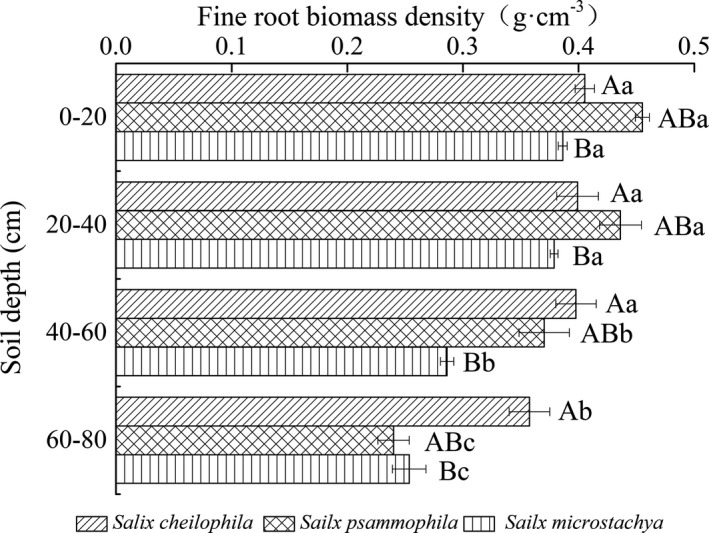
Vertical distribution of fine root biomass density in three shrubs (*S. cheilophila*, *S. psammophila,* and *S*. *microstachy*). Lowercase and capital letters indicate the significant difference of mean values of fine root biomass in different soil depth interval and fine root biomass in different shrub species, respectively, among the treatments. Bars with the same letter indicate a non‐significant difference (*p* = 0.05)

Approximately 65%~75% of fine roots were concentrated at the 0~40 cm depth interval, and decreased as soil depth increased (Figure 2). Fine root biomass density (0–80 cm) of *S. cheilophila*, *S. psammophila,* and *S*. *microstachya* was 1.56 ± 0.06, 1.50 ± 0.06, and 1.30 ± 0.03 g/cm^3^, respectively. The fine root biomass of *S. psammophila* in surface soil (0–40 cm) was significantly higher than that of *S. cheilophila* and *S*. *microstachya* (*p* < 0.05), while the fine root biomass of *S. cheilophila* in deep soil (40–80 cm) was significantly greater than that of *S. psammophila* and *S*. *microstachya (p* < 0.05; Figure 2).

Fine root extinction coefficient (0‐80cm) of *S. cheilophila*, *S. psammophila,* and *S. microstachya* was 0.9470 ± 0.0058, 0.9313 ± 0.0036, and 0.9318 ± 0.0095, respectively. Higher root extinction coefficient indicated that the distribution of *S. cheilophila* was more even and deeper in the soil than the other two.

### 3.2 Fine root decomposition

**FIGURE 3:**
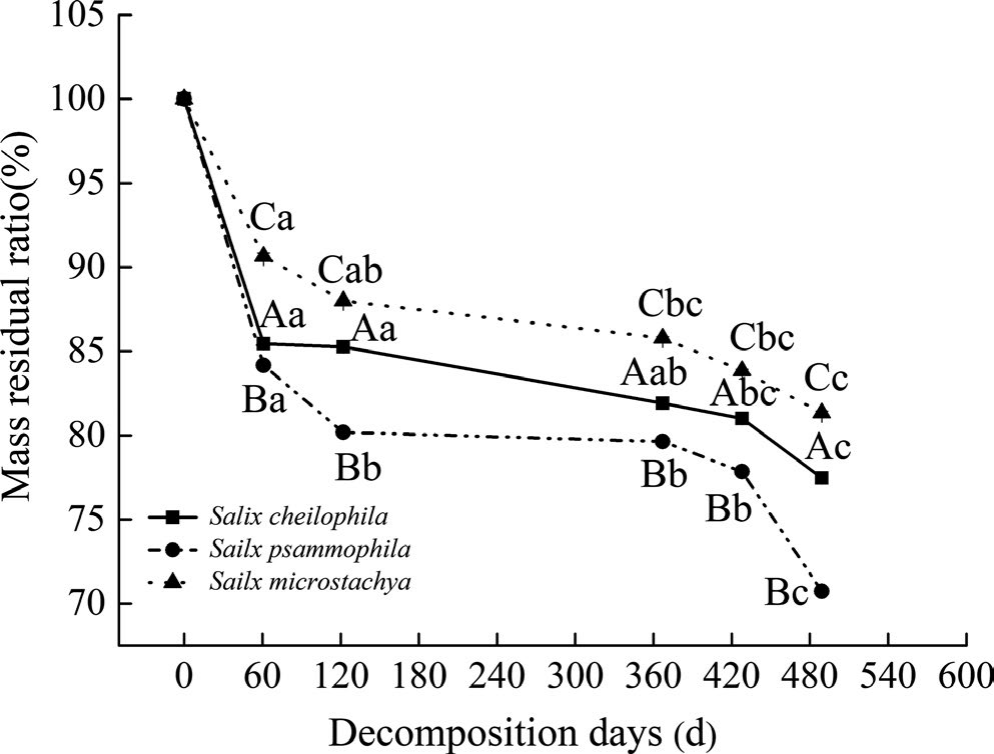
Variation of mass residual ratio of fine root for three shrubs after the decomposition of 489 days

It can be noticed from Figure 3 that the residual mass of fine root in *S. microstachya* was the smallest among the other three shrubs. Fast decomposition occurred in the initial phase (0~120 days, summer settlement), and slow decomposition was observed in the second period (120~360 days, winter settlement), followed by visible decomposition again in the last period (360~489 days, summer settlement). Mass residual ratio of fine roots was 80.18 ± 0.06% for *S. psammophila* after 60 days of decomposition, and reduced to 70.73 ± 0.12% after 489 days of decomposition.

As shown in Table 1, the time required for 50% and 95% decomposition of the three shrubs were remarkably different, which appeared as *S. psammophila* (0.101 kg kg^−1^ year^−1^) > *S. microstachya* (0.081 kg kg^−1^ year^−1^) > *S. cheilophila* (0.073 kg kg^−1^ year^−1^). The time required for 95% loss of fine roots of *S*. *cheilophila*, *S*. *psammophila,* and *S*. *microstachya* were 39.00, 27.99, and 35.95 years, respectively.

**TABLE 1:** Olson exponential regression equation of mass residual ratio of fine roots in three shrubs

Species	Decomposition coefficient/(kg kg^−1^ yr ^−1^)	Fitting parameters (a)	Fitting precision (*R* ^2^)	Measured value of mass residual ratio after 489 days (%)	Theoretical value of mass residual ratio after 489 days (%)	Time for 50% fine root decomposition (year)	Time for 95% fine root decomposition (year)
*S. cheilophila*	0.073	e^4.468^	0.85	77.46	79.00	7.58	39.00
*S. psammophila*	0.101	e^4.443^	0.93	70.73	74.24	5.24	27.99
*S. microstachya*	0.081	e^4.518^	0.92	81.34	82.22	7.49	35.95

**FIGURE 4:**
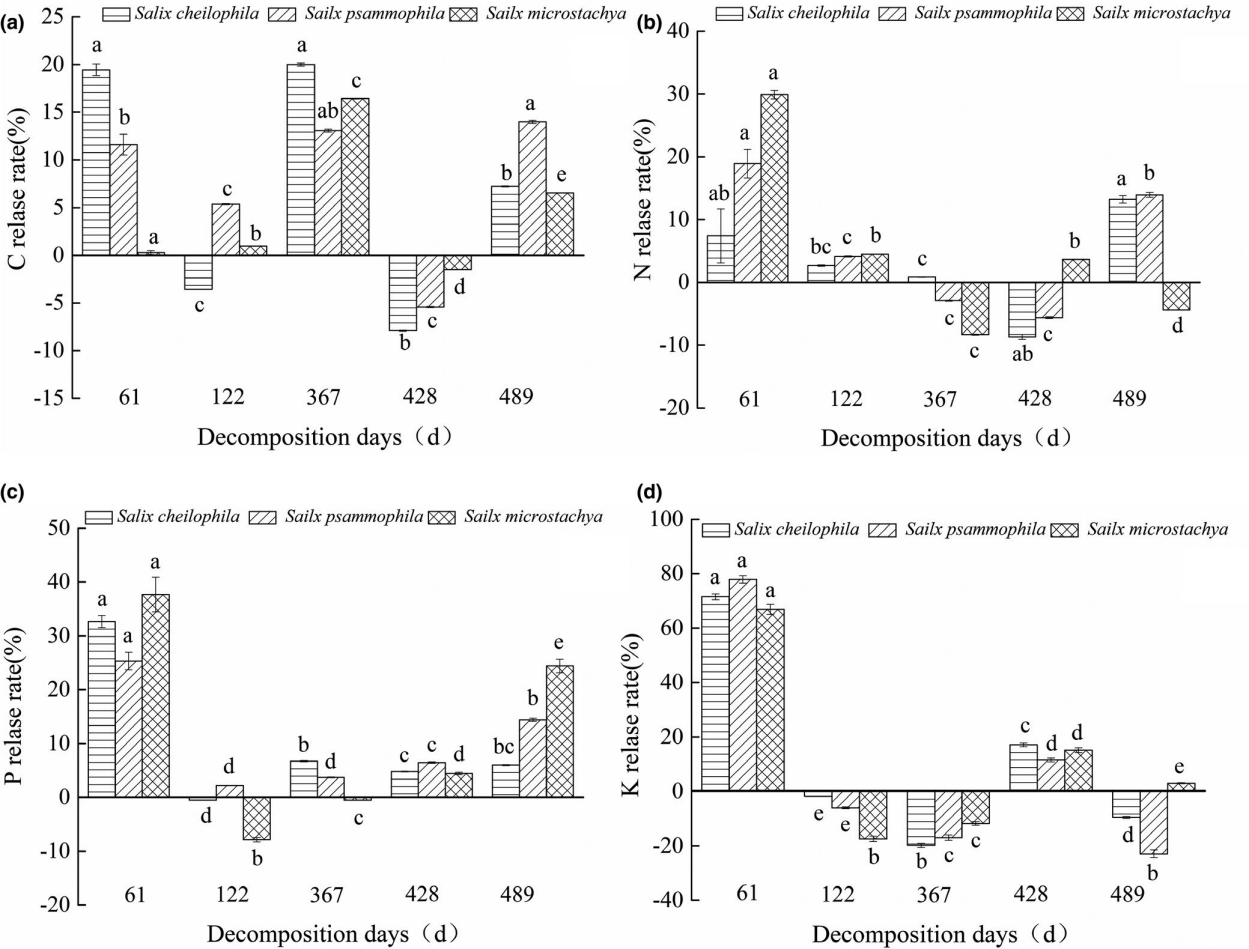
C, N, P, and K release rates (%) of fine roots (a) ~ (d) for three shrubs (*S. cheilophila*, *S. psammophila,* and *S*. *microstachy*). Lowercase letters indicate the significant difference of mean values of fine root C, N, P, and K release rates in different decomposition period of three shrubs. Bars with the same letter indicate a non‐significant difference (*p* = 0.05)

Figure 4 shows that different levels of release and enrichment of nutrients (C, N, P, and K) occurred during the whole process of fine root decomposition. Decomposition rate achieved the maximum during the first 61 days, and nutrient release rates of C for *S. cheilophila* and *S. psammophila* were 19.44% ± 0.61% and 11.60% ± 1.09%, respectively, which were markedly larger than that of *S*. *microstachya* (0.28% ± 0.20%), while N (29.90% ± 0.67%) and P (37.66% ± 3.22%) for *S*. *microstachya* were the largest within the three shrubs (*p* < 0.05). After 489 days’ decomposition, nutrient release rates of C for three shrubs were *S. psammophila* (13.98% ± 0.17%) > *S. cheilophila* (7.21% ± 0.05%) > *S. microstachya* (6.53% ± 0.01%), while N possessed a release rate of 13.22% ± 0.61% and 13.93% ± 0.39% for *S. cheilophila* and *S. psammophila*, respectively. Particularly, *S. microstachya* was enriched with N for release rate of 4.39% ± 0.04% from soil.

### 3.3 Fine root turnover and production

**FIGURE 5:**
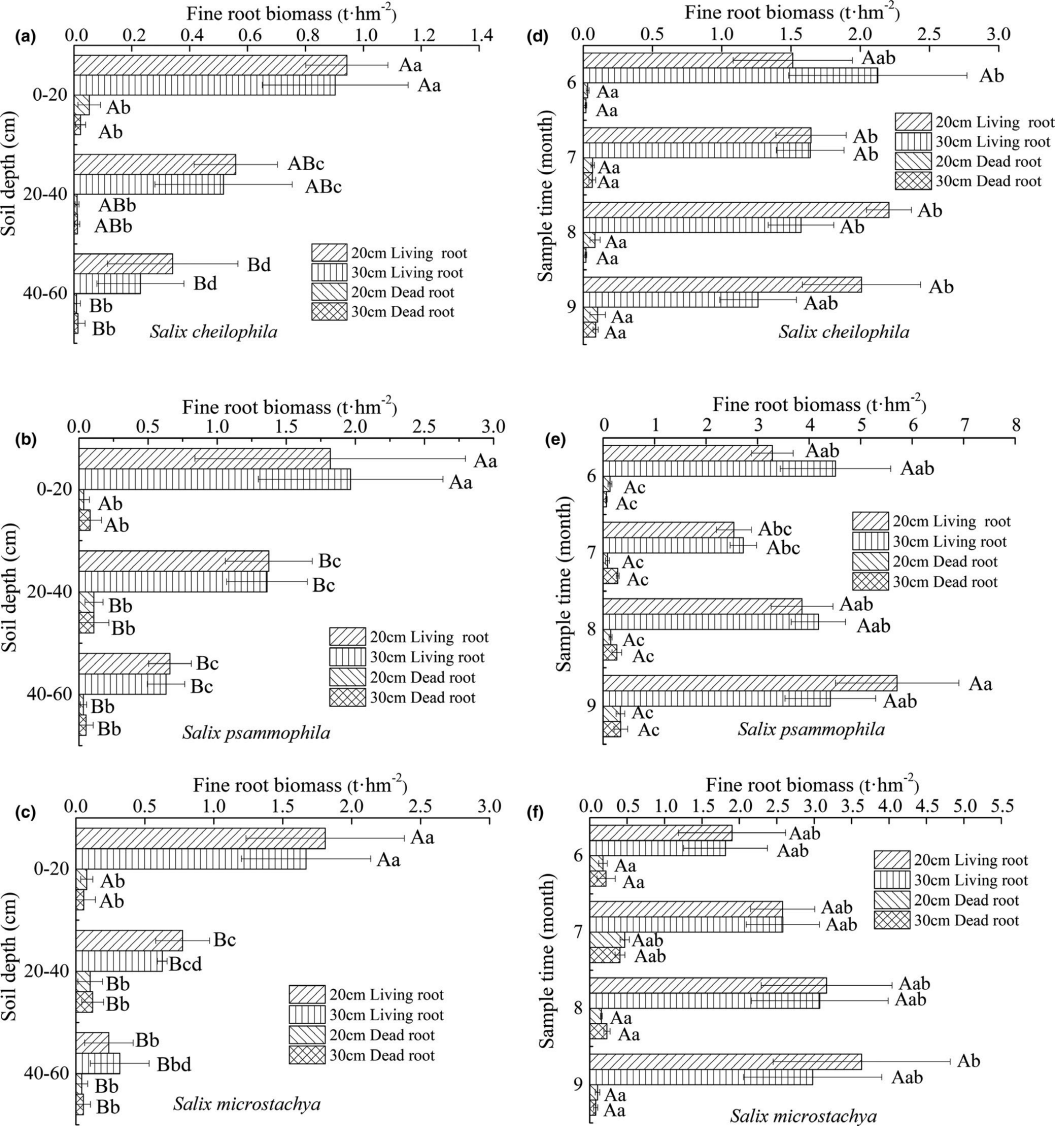
Vertical distribution (a) ~ (c) of three shrubs (*S. cheilophila*, *S. psammophila,* and *S*. *microstachy*) with different horizontal sample radius of fine root living and dead biomass (biomass of per depths represent averaged across months); different sample period (d) ~ (f) of three shrubs (*S. cheilophila*, *S. psammophila,* and *S*. *microstachy*) with different horizontal sample radius of fine root living and dead biomass (biomass of per months represent sum of biomass for all depths). Bars indicate the standard error of the means. Lowercase and capital letters indicate the significant difference of mean values of fine root living and dead biomass in different horizontal sample radius and living and dead biomass in different soil depth interval (or in different sample period), respectively. Bars with the same letter indicate a non‐significant difference (*p* = 0.05)

Figure 5 shows that fine root living and dead biomass of three shrubs had a decreasing tendency with the depth, and the biomass of living fine roots were remarkably larger than that of fine root necromass (approximately 5%~12% of the total fine root biomass; *p* < 0.05). Approximately 82%~89% of fine roots of three shrubs were concentrated at the 0~40 cm depth interval, which indicated that fine root turnover mostly occurred close to surface soil. The maximum biomass of living fine roots of *S. psammophila* (5.710 t·hm^‐2^) and *S*. *microstachya* (3.634 t/hm^2^) appeared at the sampling radius of 20 cm in September, while that happened to *S. cheilophila* in June (2.127 t/hm^2^).

**FIGURE 6:**
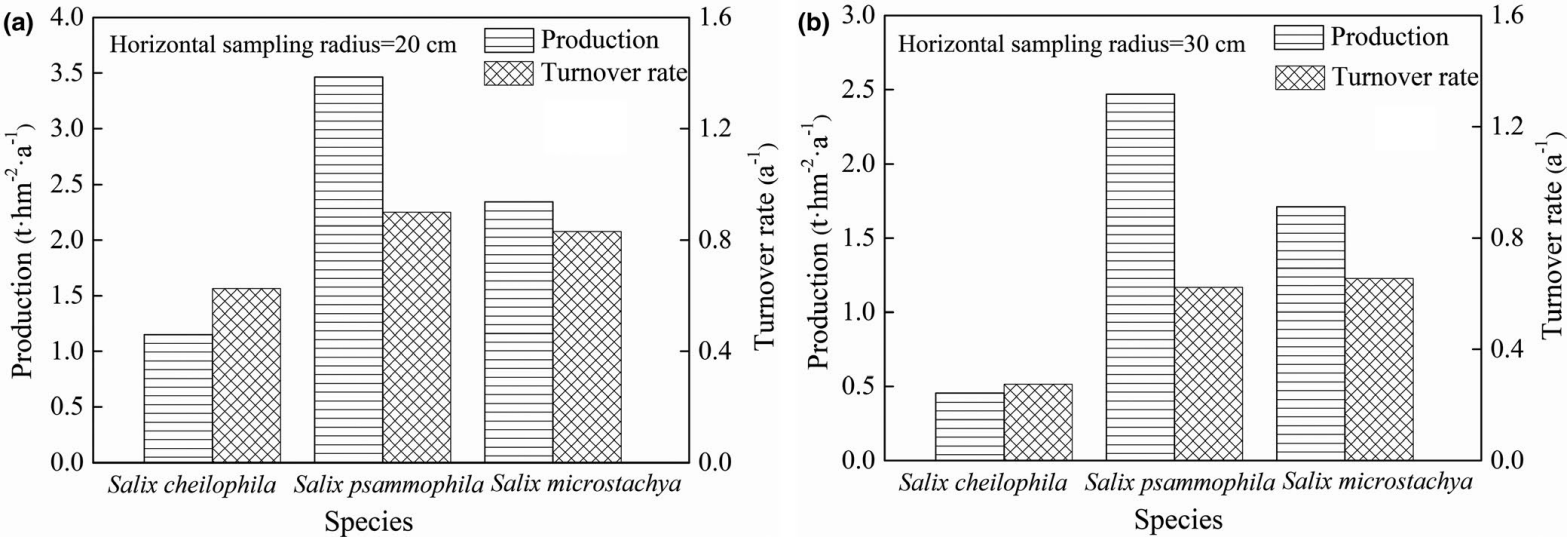
Different horizontal sample radius (R = 20 cm(a), R = 30 cm(b)); of fine root production and turnover rate for fine root biomass of three shrubs (*S. cheilophila, S. psammophila, and S. microstachy*)

Fine root production of *S. cheilophila*, *S. psammophila,* and *S. microstachya* were 1.152, 3.466, and 2.343 t hm^−2^ year^−1^, while the three had a turnover rate of 0.625, 0.900 and 0.831 yr^−1^, respectively, when the horizontal sampling radius was 20 cm (Figure 6). Fine root production and turnover rate with the horizontal sampling radius at 30 cm were remarkably smaller than that at 20 cm.

It can be noticed from Table 2 that the production of fine roots was significantly positive correlated with soil temperature (*p* < 0.05). Soil moisture was significantly positive correlated with the turnover rate of fine roots at a sampling radius of 30 cm (*p* < 0.05). There was an extremely significantly positive correlation between fine root production and living fine root biomass (*p* < 0.01). The interaction between fine root production at sample radius of 20 and 30 cm was significantly positive correlated with fine root total nitrogen (*p* < 0.05), while negative correlated with fine root total potassium.

**TABLE 2:** Correlation coefficients between fine root production, turnover rate, soil physical and chemical properties, fine root biomass, and fine root chemical element content of three shrubs (*S. cheilophila, S. psammophila, and S. microstachy)*

Soil environmental and fine root factors	20 cm Horizontal sample radius		30 cm Horizontal sample radius	
	NPP	Turnover rate	NPP	Turnover rate
Temperature	0.512*	0.174	0.543*	0.093
Humidity	0.293	0.577	0.418	0.685*
Soil Organic Carbon	0.193	−0.258	0.157	−0.295
Soil Nitrogen	0.135	−0.247	0.075	−0.322
Soil Phosphorus	0.215	0.056	0.120	−0.131
Soil Potassium	0.094	−0.062	0.053	−0.131
Living fine root biomass	0.909**	−0.048	0.841**	−0.202
Dead fine root biomass	−0.218	−0.071	0.128	0.141
Fine root total carbon	−0.406	0.154	−0.422	0.119
Fine root total nitrogen	0.067*	−0.390	0.042*	−0.109
Fine root total phosphorus	0.410	0.350	0.274	0.027
Fine root total potassium	−0.431*	0.469	−0.286*	0.664*

Note

* and ** represent *p* < 0.05 and *p* < 0.01, respectively.

### 3.4 Plantation carbon sequestration

The above‐ground biomass was remarkably higher than belowground biomass in all three *Salix* shrubs, vegetation total biomass was 20.68, 23.25 and 12.90 t/hm^2^ for *S. cheilophila*, *S. psammophila,* and *S. microstachya*, respectively (Table 3). The component, above‐ground, below‐ground, and total biomass for the *Salix* plantations stands significantly differ with the vegetation types (*p* < 0.05).

**TABLE 3:** Biomass in different components of *S. cheilophila*, *S. psammophila,* and *S. microstachya*

Components	Biomass of different plantations / t/hm^2^		
	*S. cheilophila*	*S. psammophila*	*S. microstachya*
Stem	8.14 ± 0.80^a^	9.01 ± 0.36^bc^	5.35 ± 2.97^c^
Branch	4.02 ± 0.36^a^	3.56 ± 0.37^b^	4.47 ± 2.48^b^
Leaf	1.16 ± 0.11^a^	1.09 ± 0.17^a^	1.18 ± 0.65^b^
Bark	0.91 ± 0.10^a^	0.95 ± 0.15^a^	0.62 ± 0.35^b^
Fine root, Roots ≤ 2 mm	0.57 ± 0.06^a^	0.62 ± 0.06^b^	0.15 ± 0.01^bc^
Medium root, 2 mm < Roots ≤5 mm	0.93 ± 0.10^a^	1.50 ± 0.20^ab^	0.31 ± 0.04^c^
Thick root, Roots > 5 mm	4.94 ± 0.49^a^	6.52 ± 0.19^c^	0.82 ± 0.12^ab^
Above‐ground biomass	14.23 ± 1.38^a^	14.61 ± 1.05^bc^	11.62 ± 6.46^b^
Belowground biomass	6.45 ± 0.44^a^	8.64 ± 1.39^c^	1.28 ± 0.14^bc^
Total biomass	20.68 ± 1.82^a^	23.25 ± 2.44^b^	12.90 ± 6.60^c^

Note

Values followed by different lowercase letters indicate significant differences among vegetation types according to Duncan's multiple range test (*p* < 0.05). The values are the means ± *SE* (*n* = 3).

**FIGURE 7:**
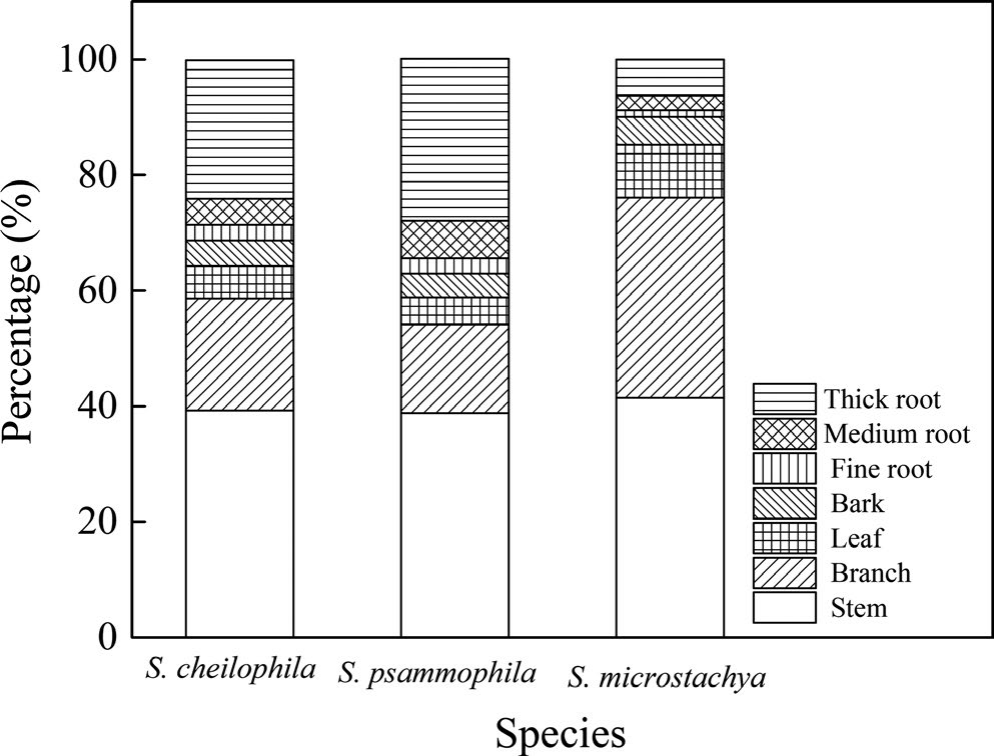
Proportion of the biomass of different components in three shrubs (*S. cheilophila, S. psammophila,* and *S. microstachy*) plantations

It is shown in Figure 7 that root was the components that accounted for the largest amount of its total biomass among the three shrubs (approximately 37% for *S. psammophila*), while *S. microstachya* posseesed the highest proportion of branch and stem (approximately 35% and 41%). The order of biomass of components for *S. cheilophila* and *S. psammophila* was similar, while that was different for *S. microstachya*.

The carbon concentration of each component of *S. cheilophila, S. psammophila,* and *S. microstachy* plantation were significantly different (*p* < 0.05; Table 4). The carbon concentrations in the components did not change significantly with the vegetation types. The carbon density of the root system of three shrubs were lower than that of the above‐ground components, and the carbon concentrations of the three *Salix* shrubs components ranged from 0.37 to 0.50 g C g^−1^.

**TABLE 4:** Biomass carbon concentrations for *S. cheilophila*, *S. psammophila,* and *S. microstachya*

Components	Carbon concentration of different plantations / gC/g		
	*S. cheilophila*	*S. psammophila*	*S. microstachya*
Stem	0.50 ± 0.01^c^	0.51 ± 0.01^c^	0.48 ± 0.01^c^
Branch	0.47 ± 0.01^b^	0.48 ± 0.01^bc^	0.49 ± 0.01^bc^
Leaf	0.47 ± 0.01^b^	0.46 ± 0.01^ab^	0.49 ± 0.01^c^
Bark	0.48 ± 0.02^bc^	0.48 ± 0.01^bc^	0.47 ± 0.01^b^
Fine root, Roots ≤ 2 mm	0.45 ± 0.01^a^	0.43 ± 0.02^a^	0.37 ± 0.01^a^
Medium root, 2 mm < Roots ≤5 mm	0.45 ± 0.01^a^	0.46 ± 0.01^ab^	0.46 ± 0.01^ab^
Thick root, Roots > 5 mm	0.43 ± 0.01^a^	0.44 ± 0.01^a^	0.44 ± 0.01^a^

Note

Values followed by different lowercase letters indicate a significant difference among the components according to Duncan's multiple range test (*p* < 0.05). The values are the means ± *SE* (*n* = 3).

The composition, above‐ground, below‐ground, and total biomass carbon densities of of *S. cheilophila, S. psammophila,* and *S. microstachy* plantation were significantly different (*p* < 0.05; Table 5). The biomass carbon densities of *S. cheilophila, S. psammophila,* and *S. microstachy* plantation were 1.42, 2.39, and 1.64 t/hm^2^, respectively. The aboveground and belowground biomass carbon density of three *Salix* shrubs showed the same trend, which showed the largest leaf aboveground and the largest fine roots belowground.

**TABLE 5:** Biomass carbon density for *S. cheilophila*, *S. psammophila,* and *S. microstachya* plantation (t/ha)

Components	Biomass carbon density of different plantations / t/hm^2^		
	*S. cheilophila*	*S. psammophila*	*S. microstachya*
Stem	0.16 ± 0.07^a^	0.18 ± 0.08^b^	0.10 ± 0.03^bc^
Branch	0.08 ± 0.02^b^	0.07 ± 0.02^a^	0.09 ± 0.03^b^
Leaf	0.54 ± 0.21^ac^	0.50 ± 0.19^ac^	0.56 ± 0.23^a^
Bark	0.02 ± 0.01^b^	0.02 ± 0.01^a^	0.01 ± 0.01^b^
Fine root, Roots ≤ 2 mm	0.51 ± 0.16^c^	1.48 ± 0.34^bc^	0.86 ± 0.25^a^
Medium root, 2 mm < Roots ≤5 mm	0.02 ± 0.01^b^	0.03 ± 0.01^a^	0.01 ± 0.01^b^
Thick root, Roots > 5 mm	0.09 ± 0.02^b^	0.11 ± 0.07^ab^	0.01 ± 0.01^b^
Above‐ground biomass	0.80 ± 0.23^a^	0.77 ± 0.21^bc^	0.76 ± 0.20^ab^
Belowground biomass	0.63 ± 0.15^ac^	1.62 ± 0.73^bc^	0.88 ± 0.27^ab^
Total biomass	1.42 ± 0.68^a^	2.39 ± 1.18^c^	1.64 ± 0.75^ab^

Note

Values followed by different lowercase letters indicate significant differences among the components according to Duncan's multiple range test (*p* < 0.05). The values are the means ± *SE* (*n* = 3).

The total carbon sequestration of plant components, litter and soil for three shrubs plantations were estimated 9.53, 6.77, and 4.50 t hm^−2^ year^−1^ in *S. psammophila*, *S*. *microstachya*, and *S. cheilophila* plantations, respectively (Table 6). The ratio of fine roots’ carbon sequestration to plantations’ total carbon sequestration amount of three shrubs plantations was *S. psammophila* (15.5%) > *S*. *microstachya* (12.7%) > *S. cheilophila* (11.5%).

**TABLE 6:** Carbon sequestration of plant components, litter and soil for *S. psammophila, S. microstachya*, and *S. cheilophila* plantations

Plantation	Carbon sequestration of plant components/ t hm^−2^ year^−1^	Carbon sequestration of litter/ t hm^−2^ year^−1^	Carbon sequestration of soil/ t hm^−2^ year^−1^	Total Carbon sequestration / t hm^−2^ year^−1^
*S. cheilophila*	1.42	0.46	2.62	4.50
*S. psammophila*	2.39	1.96	5.18	9.53
*S. microstachya*	1.64	0.14	4.99	6.77

## 4 DISCUSSION

### 4.1 Fine roots biomass vertical distribution patterns

Approximately 65%~75% of fine roots of *S. cheilophila, S. psammophila,* and *S. microstachya* were concentrated at the 0~40 cm depth interval (Figure 2). Fine root distribution patterns of *S. cheilophila, S. psammophila,* and *S. microstachya* were like those in many other drylands that a higher content of fine roots existed near the surface of soil (Chang et al., 2012; Lai et al., 2016). Our study results showed that fine roots of three *Salix* shrubs were mainly distributed in the surface soil. That might be adaptation strategies of plants, which had reduced the transportation cost of vegetation organic matter, and improved the rapid absorption of water and nutrients infiltrated into the surface soil by vegetation (Chang et al., 2012; Huang et al., 2008; Oppelt et al., 2005). In addition, the shallow root distribution of the three shrubs might be enhanced by the perennial gale climate in Gonghe Basin. Vertical patterns of fine root extinction coefficient varied no remarkably among shrub species. In this study, differences in vertical distribution of fine roots among shrub species suggested different adaption mechanisms exist among them when environment resources (water and nutrients) were limited. Compared with the other two shrubs, *S. cheilophila* had stronger adaptability. The fine roots of *S. cheilophila* were distributed much deeper, which indicated that *S. cheilophila* not only had advantages in soil consolidation, but also could utilize water in deep soil when resources were scarce in drylands (Yu & Jia, 2014).

### 4.2 Effects of environmental factors on patterns of fine root decomposition

After 489 days of fine root decomposition, the mass residual ratio of fine root of *S. psammophila* was the lowest (70.73%), followed by *S. cheilophila* (77.46%) and *S. microstachya* (81.34%; Figure 3). In the early stage of decomposition (0~120 days, summer settlement), rapid eluviation of carbohydrates and other soluble substances had be led by environmental factors, such as soil temperature, soil moisture content, and decomposition substrate. Soil temperature enhancing significantly had interacted with throughfall reduction in accelerating the decomposition rate of fine roots, while soil temperature enhancing significantly had elevated carbon release of fine roots by 18.1% (Liu et al., 2017). In the second period (winter settlement), slow decomposition was observed, and the decomposition process had affected by the low temperature and biological action that exhausted soluble compounds. Along with soil temperature decreasing in winter settlement, fine roots decomposition rate slowed down. What's more, N and P had immobilized into fine roots in winter settlement, the labile carbohydrates had decreased over time, with insoluble substances such as lignin and cellulose remaining, which had slowly degraded by microorganisms. (Xu et al., 2013). However, in the following growing season, the decomposition rate had raised with the increase of soil temperature and water content (Tu et al., 2015; Wang et al., 2011).

In this study, the fitting of Olson exponential decay model indicated that the fine roots total decomposition time of *S. cheilophila, S. psammophila* and *S. microstachya* are 39.00, 27.99 and 35.95 years, respectively (Table 1). The decomposition rate was remarkably lower than that of other sandy regions, such as *Caragana korshinskii* and *S. psammophila* planted in the desert area in Ningxia Mu Us Desert which had possessed a T_95_ of 8.52 and 18.80 years, respectively (Lai et al., 2016). This case had related to climate, soil temperature, microbial biomass C and vegetation types (Bergner et al., 2004). In our study area, the temperature was lower than other sandy areas, with fewer soil microbial species and lower soil microbial activity. After 489 days’ decomposition, fine‐root C loss of *S. cheilophila, S. psammophila,* and *S. microstachya* were 7.21% ± 0.05%, 13.98% ± 0.17%, and 6.53% ± 0.01%, respectively (Figure 4). That caused by soil microbial communities, which had controlled the decomposition of fine roots and thus regulate C mineralization, had primarily affected by vegetation types (Clemmensen et al., 2013). Additionally, the decomposition rate of fine roots had depended more on soil microbial biomass carbon, which accounted for 33.1% (*p* < 0.05) of the variation in decomposition rate of root (Liu et al., 2017). A 10‐year root decomposition experiment in 21 sites from seven biomes had found that root‐litter decomposition was slowest in the cold, arid regions, and fastest in the warm, moist tropical forests, and the climatic decomposition index (CDI) which incorporated seasonality in temperature and moisture had been used as potential predictors of root decomposition rates, the CDI for aird grasslands was <50% that of the humid grassland (Parton et al., 2007). Nutrients (N, P, and K) release rates of three shrubs achieved the maximum, and C release rate was high during the whole process of fine root decomposition (Figure 4). The elements of dead root with higher initial concentration were easily to had been released in forest (Vogt et al., 1986), N and P of plant residue decomposition had been released into the soil in the form of minerals, when the concentration of N and P in the organism had reached critical values (Manzoni et al., 2008). Our study was conducted in an alpine sandy land in Qinghai at an altitude of 2,871~3,870 m that lower activities of cellulose and lignin degrading enzymes had occurred in soil and microbial activity (He et al., 2019; Parton et al., 2007). Therefore, fine root decomposition rate differed distinctly even for the same species as the study area varied.

### 4.3 Effects of turnover on fine root production in three shrubs

Fine root production at different sample times and soil depths of *S. psammophila* were remarkably higher than that of *S. cheilophila* or *S. microstachya*. The highest amount of necromass occurred close to surface (0~40 cm; Figure 5), which had been attributed to the fact that fast water evapotranspiration rate in semi‐arid and arid regions with the topsoil generally or periodically dry (Gwenzi et al., 2011; Zhang, Li, et al., 2009). Nevertheless, the fine roots of different plant species had responded to temporal changes of the topsoil with different adaptation strategies (Ward et al., 2013).

Some studies had showed that fine root production had different vertical distribution patterns in different soil profiles due to heterogeneity of soil nutrients and water, however, the root distribution patterns had showed certain similarities under the same site conditions (Wang et al., 2014). In our study, fine root turnover rates of them were *S. psammophila* (0.90 yr^−1^), *S. microstachya* (0.83 yr^−1^) and *S. cheilophila* (0.63 yr^−1^), respectively (Figure 6), which were within the thresholds of the study of the turnover rate of fine roots in global vegetation (Jackson et al., 1997). Previous studies had indicated that the fine root turnover rate of vegetation in arid and semi‐arid desert ecosystem was higher than that in wet area (Lukac & Godbold, 2010), for adapting to the environmental stress of drought and water shortage, and further obtaining more soil resources (Huang et al., 2008). In addition, fine root turnover rate of vegetation had been controlled by its own genetic characteristics (Pei et al., 2012). In our study, soil moisture of three shrubs exerted a significant effect on fine root turnover, while soil temperature had a significant positive correlation with fine root production (Table 2). Some studies had also showed that higher turnover rate was obtained due to the increase in temperature and rainfall events during summer and late autumn period, which had led to faster growth of fine roots (Montagnoli et al., 2019). The comparison in soil environmental factors showed that the difference of fine root production could be explained by living fine root biomass to some extent. Other studies had also founded that fine root production had rose with the increase of living root biomass (Finér et al., 2011; Li et al., 2003).

### 4.4 Contribution of fine root dynamic activities to carbon sequestration of plantation

The biomass quantification of plants had been recognized as a crucial step in calculation of forest biomass and carbon stocks (Pilli et al., 2006). Vegetation had continuously absorbed CO_2_ during the photosynthesis and had subsequently stored the resulting carbohydrates in their biomass in shrub plantations, which had positive impacts on the forest ecosystem and nutrient cycling (Li et al., 2018). However, each tree species had its own biomass and carbon sequestration capacity (Deng & Shangguan, 2017). In our results, the biomass carbon density of *S. cheilophila, S. psammophila,* and *S. microstachya* plantations were 1.42 ± 0.68*,* 2.39 ± 1.18, and 1.64 ± 0.75 t/hm^2^ *,* respectively (Table 5)*,* which had been lower than the Chihuahuan desert ecosystem in North America biomass carbon density of 3.2 t/hm^2^ (Havstad et al., 2006)*,* and Sonoran desert biomass carbon density of 6.4 t/hm^2^ (Búrquez et al., 2010), the global average desert biomass carbon density of 7.0 t/hm^2^ (Houghton et al., 2009), respectively. Although the biomass carbon density of *S. cheilophila, S. psammophila,* and *S. microstachya* plantations in the alpine sandy land was lower than other deserts, these three *Salix* plantations were also important carbon pools due to the large distribution of the alpine sandy area. And if all of the sandy land was restored, it would have great potential for carbon sequestration.

Carbon sequestration and distribution varied significantly in three shrub plantation. *S. psammophila* had the highest total carbon sequestration, followed by *S. microstachya* and *S. cheilophila* (Table 6). The results had been relatively lower than the result in semi‐arid region (Lai et al., 2017). Other studies had showed that the carbon sequestration of plantation came from the photosynthesis of branches and leaves of vegetation, the continuous growth, death, and decomposition of roots, which had further increased carbon input to soil in degraded drylands (Hobbie et al., 2010; Li et al., 2018; Yüksek & Yüksek, 2011). A high content of total fine root biomass had supported the return of C to the soil, and up to 70% of SOC sequestration had been derived from roots (Clemmensen et al., 2013; Finér et al., 2019). Our results showed the ratio of fine roots’ carbon sequestration to plantations’ total carbon sequestration amount were 15.5%, 12.7%, and 11.5% for *S. psammophila*, *S. microstachya,* and *S. cheilophila,* respectively. Some studies had reported that sand‐fixing vegetation in desert ecosystem often had a higher ratio of above‐ground and below‐ground biomass, which had indicated that the dynamic activities of root system had an important impact on SOC content (Lai et al., 2016). Another study had also showed that total belowground C input from fine root litter production was 1.4‐fold greater than that of above‐ground from leaf litter production, although fine root biomass had accounted for a small proportion of total vegetation biomass (Ding et al., 2019). However, in the tropical and subtropical forest ecosystem, the largest contribution rate of carbon content had been the litter on the ground (Tang et al., 2013). These results had suggested that the fine root seasonal dynamics of shrubs in different ecosystems contributed to carbon sequestration at different rates.

## 5 CONCLUSIONS

Fine root dynamic activities of three *Salix* shrubs contributed remarkably to total carbon sequestration of plantations. Biomass carbon density for three *Salix* plantations ranged from 1.42 to 2.39 t/hm^2^, which showed that three *Salix* plantations in alpine sandy land are an important carbon pool. In addition, fine root biomass carbon density for the three shrub plantations varied significantly. And fine root biomass carbon density for *S. psammophila* reached the largest among the three plantations, which was 1.48 t/hm^2^, accounting for the ratio of 62% of the plantation total‐biomass carbon density. The results indicated that the root system of *S. psammophila*, especially the fine roots, is very developed, which is conducive to soil retaining, soil water transportation and carbon sequestration. *S. psammophila* might be a better tree species for carbon sequestration of plantation and desertification control in alpine sandy areas.

The carbon input from the fine roots of the three shrub plantations through decomposition and turnover into the plantations accounts for 11.5%–15.5% of total carbon sequestration of plantations. Therefore, the fine roots dynamics must be considered for long‐term carbon pool estimations in three *Salix* plantations, otherwise the total carbon sequestration of plantations would be underestimated.

## CONFLICT OF INTEREST

None declared.

## AUTHOR CONTRIBUTIONS

**Lingxianzi He:** Conceptualization (equal); Data curation (equal); Formal analysis (equal); Investigation (equal); Methodology (equal); Software (equal); Writing‐original draft (equal). **Zhiqing Jia:** Funding acquisition (equal); Project administration (equal); Resources (equal); Writing‐review & editing (lead). **Qingxue Li:** Data curation (supporting); Formal analysis (supporting); Software (supporting); Supervision (supporting). **Youyan Zhang:** Investigation (supporting); Methodology (supporting); Validation (supporting); Visualization (supporting). **Rina Wu:** Data curation (supporting); Investigation (supporting); Software (supporting); Visualization (supporting). **Jie Dai:** Methodology (supporting); Visualization (supporting). **Ya Gao:** Investigation (supporting); Software (supporting).

## Data Availability

Archived data are available in the Dryad repository (https://doi.org/10.5061/dryad.mpg4f4qz1).
